# Distinguishing true from pseudo-absent styloid process: a case-prompted critical analysis

**DOI:** 10.1007/s00276-026-03842-w

**Published:** 2026-02-25

**Authors:** Mugurel Constantin Rusu, Vladimir Ioan Zamfirescu, Răzvan Costin Tudose

**Affiliations:** Division of Anatomy, Department 1, Faculty of Dentistry, “Carol Davila” University of Medicine and Pharmacy, 8 Eroilor Sanitari Blvd., 050474 Bucharest, Romania

**Keywords:** Tympanohyal, Tympanic plate, Stylohyoid complex, Cone-beam computed tomography, Temporal bone, Reichert's cartilage

## Abstract

The styloid process (SP) of the temporal bone is well-documented for its morphological variability, yet the distinction between true absence and pseudo-absence remains poorly defined in the literature. This study critically examines published reports of SP absence and proposes clear terminological criteria to differentiate these entities. True absent SP denotes aplasia of both tympanohyal and stylohyal segments, whereas pseudo-absent SP indicates a hypomineralised tympanohyal concealed beneath the vaginal process of the tympanic plate. Analysis of the literature indicates that previous studies using CT, panoramic radiography, and dry skull analysis have frequently conflated these variants, with some authors misidentifying the inferiorly projecting vaginal process as a duplicate or short SP. This critical reappraisal was prompted by a cone-beam computed tomography (CBCT) case demonstrating bilateral pseudo-absent SPs in a 39-year-old female, in which low-density tympanohyal segments (10.5 mm) were entirely masked by extensively developed vaginal processes (15.5 mm). To our knowledge, this represents the first CBCT-documented case of this morphology and only the third reported evidence, following dry-skull observations. Accurate differentiation between true and pseudo-absent SP has implications for radiological interpretation, surgical planning, and understanding stylohyoid complex development.

## Introduction

The styloid process (SP) of the temporal bone projects anteroinferiorly into the parapharyngeal space. It varies markedly in length and may be composed of multiple segments that articulate via synchondroses. The stylohyoid ligament attaches to the lesser horn of the hyoid and may calcify, forming a rigid connection with the hyoid bone [3].

The stylohyoid complex comprises the SP, the lesser hyoid horn, and the intervening stylohyoid ligament, forming a continuous osteoligamentous chain that connects the skull base to the hyoid apparatus [2]. Embryologically, this complex derives from the second pharyngeal arch and is classically described as consisting of four components: the tympanohyal segment, which gives rise to the proximal base of the SP; the stylohyal segment, which develops into the main body of the SP; the ceratohyal (keratohyal) segment, which forms the stylohyoid ligament; and the hypohyal segment, which matures into the lesser hyoid horn [2, 10]. Different ossification patterns of the stylohyoid chain may occur [4].

At the skull base, the SP arises from the inferior aspect of the temporal bone, anterior to the stylomastoid foramen and in proximity to the carotid canal and jugular foramen. The tympanic part forms the bony wall of the external acoustic meatus; its inferior rim (tympanic plate) may develop a tubal process anteriorly and a vaginal process posteriorly. The vaginal process creates a sheath around the SP base and can extend inferiorly, potentially masking an incompletely mineralised tympanohyal on three-dimensional renderings.

In this report, true absence of the SP refers to aplasia of both the tympanohyal (proximal/base) and stylohyal (shaft) segments. Pseudo-absence refers to an apparent missing SP on surface renderings caused by a hypomineralised or short tympanohyal concealed within the vaginal process sheath, with or without absence of the stylohyal. The tympanic plate is the inferior extension of the tympanic part of the temporal bone, bordering the external acoustic meatus; the vaginal process projects inferiorly and encases the SP base.

Because CBCT is widely used in dentomaxillofacial practice and routinely captures the skull base, recognising this pitfall is clinically relevant for accurate reporting and for avoiding overestimation of SP aplasia prevalence. We present a CBCT-documented bilateral pseudo-absent configuration and use it to critically reassess published “absent SP” reports under explicit anatomic criteria.

## Materials and methods

Cone-beam computed tomography (CBCT) imaging data were obtained from a 39-year-old Caucasian female of Romanian nationality during a routine dental status examination. The patient provided written informed consent for the research use of her anonymised data, in accordance with the Declaration of Helsinki.

The CBCT scan was acquired using a Planmeca imaging system (Planmeca Oy, Helsinki, Finland). DICOM datasets were imported into Horos (Horos Project, Annapolis, MD, USA) for three-dimensional volume rendering and multiplanar reconstruction. The skull base was systematically evaluated in axial, coronal, and sagittal planes. The anatomical assessment used established landmarks to identify the styloid process, specifically its emergence from the inferior temporal bone anterior to the stylomastoid foramen. The complete stylohyoid chain, including potential calcification of the stylohyoid ligament and lesser hyoid horn, was also evaluated.

## Results

**Fig. 1:**
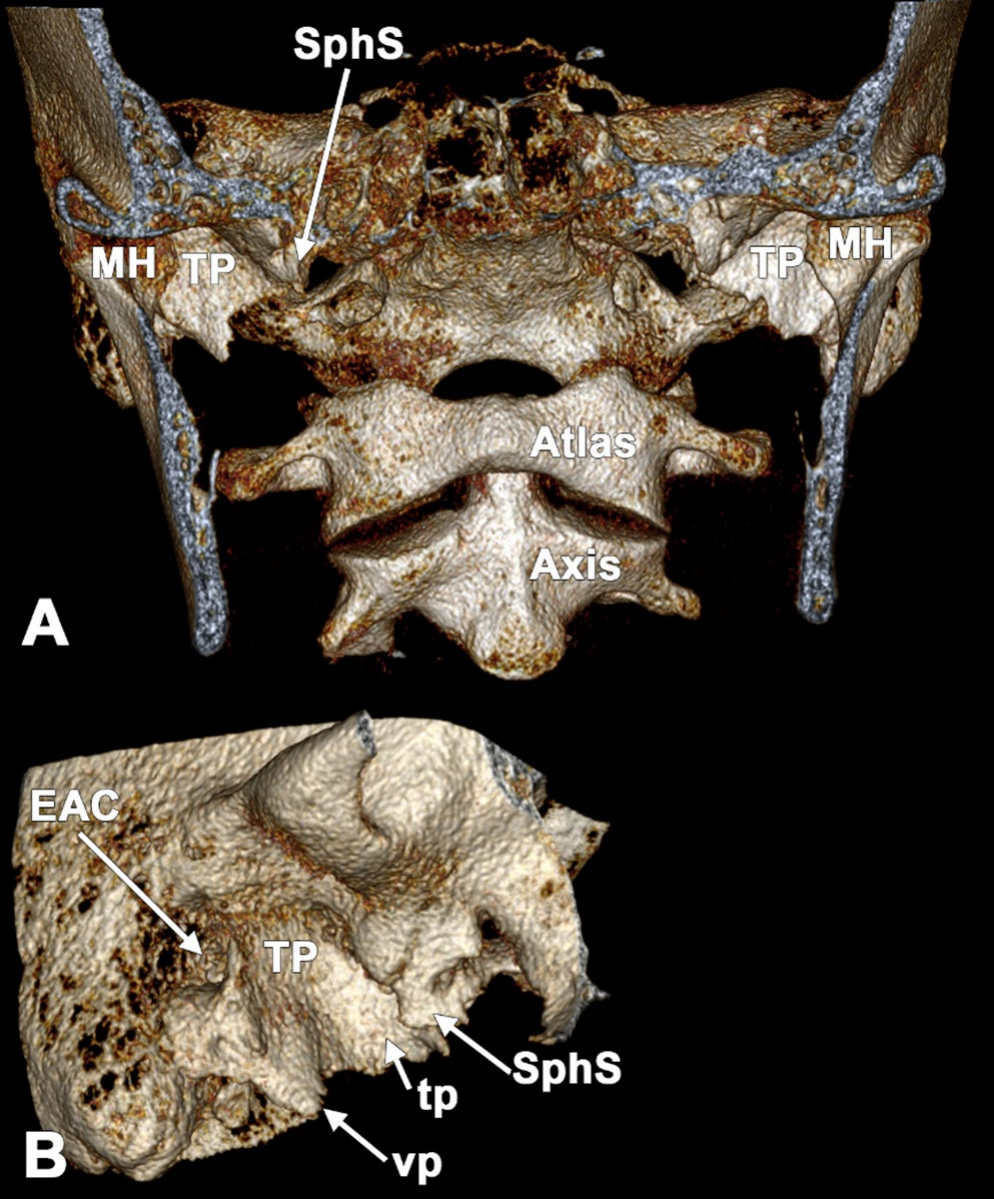
Bilateral false absent styloid processes appear on three-dimensional renderings as true absent processes; a poorly ossified tympanohyal is hidden beneath the vaginal process of the tympanic plate. Three-dimensional volume renderings. **A** anterior view; **B** lateral view of the right tympanic plate. EAC: external auditory canal; MH: mandibular head; SphS: sphenoidal spine; TP: tympanic plate; tp: tubal process; vp: vaginal process

**Fig. 2:**
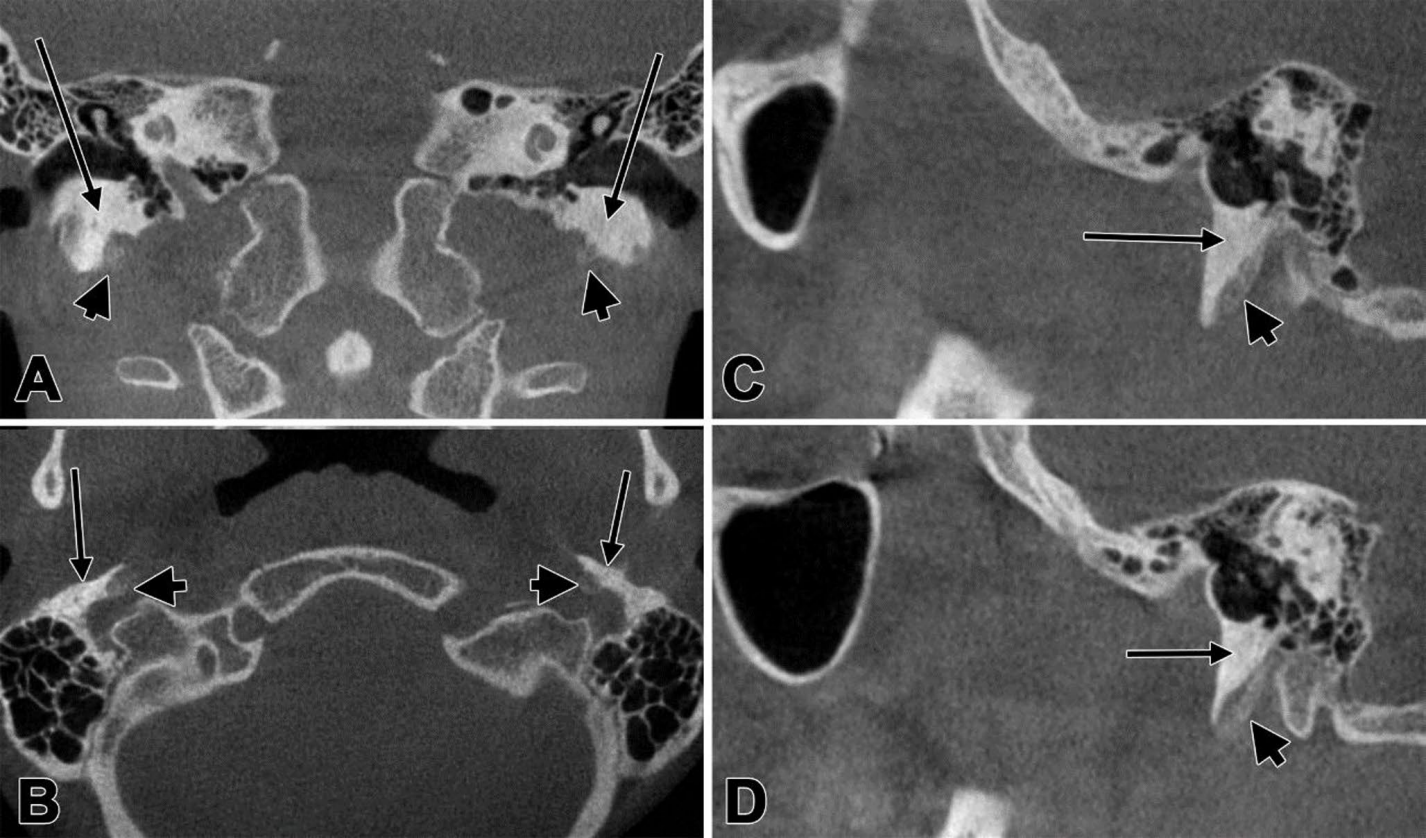
Bilateral CBCT evidence of low-density tympanohyal segments (arrowheads) beneath the vaginal processes of the tympanic plates (arrows). **A** coronal slice, anterior view; **B** axial slice, inferior view; **C** Sagittal slice, left side, lateral view; **D** Sagittal slice, right side, medial view

On careful inspection of CBCT slices, the SPs were bilaterally absent inferior to the tympanic plates (Fig. 1). However, low-density tympanohyals with cortical shells and trabecular patterns were found deep to each tympanic plate (Fig. 2). The inferior margins of the tympanic plates had inferior projections that could have been mistaken for SPs.

The tympanohyal segments exhibited markedly low attenuation compared to adjacent highly mineralised bone, and critically, these segments measured only 10.5 mm bilaterally, failing to extend beyond the distal margins of the extensively developed tympanic plates, which measured 15.5 mm. Consequently, the low-density tympanohyal segments remained anatomically concealed within the tympanic plate sheaths, rendering them effectively invisible without targeted evaluation. The tympanohyal segments displayed asymmetric but consistently anterior sagittal angulation: 34° on the left and 26.5° on the right. This anterior inclination, combined with the poor mineralisation and anatomical masking by the tympanic plates, reinforced their pseudo-absent appearance on standard review.

Both tympanic plates were markedly developed and contained air cells. The plates were symmetrically elongated, with maximal vertical extents of 15.5 mm bilaterally. Each tympanic plate typically projected two triangular processes. The anterior one was the tubal process with a tip reaching inferomedially to the tip of the sphenoidal spine. The posterior one was the vaginal process, which projected inferiorly, over the hypoossified tympanohyal. The tip of the vaginal process reached anterior to the region where an ossified stylohyal would typically be expected.

## Discussion

The styloid process (SP) originates from Reichert’s cartilage [9]. Ossification begins perinatally at the base (tympanohyal), followed by development of the shaft (stylohyal) during the third or fourth year of life [9]. Because segmental growth and fusion are highly variable, “absence” of the SP does not represent a single entity; instead, two phenotypes are recognised: pseudo-absence, in which the tympanohyal is mineralised but the stylohyal is aplastic, and true absence, defined as complete agenesis of both segments [1, 9].

A major interpretive challenge in this region is the vaginal process of the tympanic plate. Owing to its inferior orientation, it can be mistaken for a short styloid process or even interpreted as a duplicated styloid origin [9, 10]. Commonly used morphometric conventions amplify this pitfall: styloid length is often measured from the cleft between the vaginal process and the apparent styloid base [7]. While practical, this methodological starting point may bypass evaluation of the tympanohyal itself, thereby preventing reliable differentiation between true agenesis and false (apparent) absence [7].

A critical appraisal of the literature (Table 1) indicates that imaging constraints and inconsistent terminology have contributed to recurrent misclassification. MacDonald-Jankowski (2001), using panoramic radiographs, reported SP absence in 3.4–3.7% of populations but defined absence primarily as non-visualisation (“not visible”) without developmental confirmation, a criterion vulnerable to projectional and superimposition effects [6]. In a related vein, Başekim et al. (2005) described the “partial absence” of the proximal segment but misapplied the term “ceratohyal” [2], which refers to the stylohyoid ligament rather than the osseous styloid complex. Additional reports appear to have misidentified adjacent tympanic structures: Ramadan et al. (2007) and Onbas et al. (2005) interpreted the vaginal process or elongated inferior tympanic spines as “duplicate” or “double” styloid origins, thereby converting a known anatomic variant into a presumed anomaly [9, 10].

**Table 1:** Concise critical evaluation of studies of absent styloid processes

References	Method	Key findings	Critical evaluation/pitfalls
Frommer [5]	Dissection	No true absence found; SP often obscured by vaginal process	Highlighted the anatomical importance of muscle origins on the vaginal process
MacDonald-Jankowski [6]	Radiography	3.4–3.7% absence in Chinese/British samples	"Absence" is based on visibility; there is no evidence of ossification patterns
Onbas et al. [9]	CT	2.5% total absence; 20% incomplete ossification	Misidentified long inferior tympanic spines as duplicate SPs
Başekim et al. [2]	CT	4 cases of complete absence; 14 cases of partial absence	Terminological error: confused proximal (tympanohyal) with distal (ceratohyal) segments
Ramadan et al. [10]	CT	Reported non-detected ossification in 5 SPs	Misidentified the vaginal process as a "double proximal origin"
Zarandy et al. [11]	Clinical	Bilateral absence linked to Michel aplasia	Lacked presentation of imaging/anatomical evidence for SP absence
Barnes [1]	Atlas	Defined true vs. pseudo-absence variants	Clarified that true absence requires both tympano- and stylohyal aplasia
Natsis et al. [8]	Dry Skulls	3 cases of true bilateral absence; 6.04% "invisible" SPs	Identified "invisible" SPs as mineralised tympanohyals hidden by the vaginal process
Magat and Ozcan [7]	Morphometry	Measurements begin at the point where SP leaves the tympanic plate	Methodology fails to distinguish between true and false (hidden) absence

Dissection and dry-skull studies further underscore how frequently concealment is mistaken for absence. Frommer (1974) identified no true absence in 241 dissections, noting that the SP may be present but “obscured laterally” by the vaginal process [5]. Natsis et al. (2015) corroborated this mechanism in dry skulls, describing “invisible” SPs (6.04%) concealed by the vaginal process, and documented rare cases of true bilateral absence associated with compensatory changes of the tympanic plate [8]. Collectively, these findings emphasise that segment-level assessment and multiplanar imaging review are essential to avoid conflating developmental agenesis with morphological misinterpretation [7, 9, 10].

Studies of SP morphometry use the vaginal process as a reference point. Measurements of the SP were started where it leaves the tympanic plate of the temporal bone, using the cleft between the vaginal process and styloid base as a landmark [7]. With this methodology, a present or absent tympanohyal is ignored; thus, a false-absent SP cannot be distinguished from a true absence.

In summary, an apparent “absent” SP on three-dimensional renderings should not be equated with aplasia without multiplanar confirmation of the tympanohyal region deep to the tympanic plate. In cases with a well‑developed vaginal process, targeted inspection of thin slices and appropriate windowing is required to identify a hypoossified tympanohyal that the tympanic plate sheath may completely conceal. Applying explicit criteria for true versus pseudo-absence improves diagnostic consistency and supports more accurate epidemiological estimates of SP variants.

## Conclusions

We propose that true absence of SP denotes aplasia of both the tympanohyal and stylohyal segments. In contrast, a pseudo-absent SP indicates a hypomineralised tympanohyal concealed beneath the vaginal process of the tympanic plate. The inferiorly projecting vaginal process represents a critical diagnostic pitfall, potentially mistaken for a short or duplicate SP. Previous studies have likely overestimated the prevalence of true absence by failing to distinguish between these entities. Targeted evaluation deep to the tympanic plate using appropriate CT or CBCT windowing is essential for accurate diagnosis. Consistent terminology will improve epidemiological accuracy and understanding of stylohyoid complex development.

## Data Availability

The datasets used and analysed during the current study are available from the corresponding author upon reasonable request.
